# PPR647 Protein Is Required for Chloroplast RNA Editing, Splicing and Chloroplast Development in Maize

**DOI:** 10.3390/ijms222011162

**Published:** 2021-10-16

**Authors:** Yan Zhao, Wei Xu, Yongzhong Zhang, Shilei Sun, Lijing Wang, Shiyi Zhong, Xiangyu Zhao, Baoshen Liu

**Affiliations:** 1 State Key Laboratory of Crop Biology, College of Agronomy, Shandong Agricultural University, Tai’an 271018, China; zhaoyan183culb@163.com (Y.Z.); xwnxn2016@163.com (W.X.); zhangyz2005111@163.com (Y.Z.); sunslei1990@163.com (S.S.); 18353700697@163.com (L.W.); jingxizuxue@126.com (S.Z.); 2 State Key Laboratory of Crop Biology, College of Life Sciences, Shandong Agricultural University, Tai’an 271018, China; zhxy@sdau.edu.cn

**Keywords:** pentatricopeptide repeat protein, maize, albino-lethal, RNA editing, RNA splicing, chloroplast

## Abstract

Chloroplasts play an essential role in plant growth and development. Any factors affecting chloroplast development will lead to abnormal plant growth. Here, we characterized a new maize mutant, *albino seedling mutant 81647* (*as-81647*), which exhibits an entirely albino phenotype in leaves and eventually died before the three-leaf stage. Transmission electron microscopy (TEM) demonstrated that the chloroplast thylakoid membrane was impaired and the granum lamellae significantly decreased in *as-81647*. Map-based cloning and transgenic analysis confirmed that *PPR647* encodes a new chloroplast protein consisting of 11 pentratricopeptide repeat domains. Quantitative real-time PCR (qRT-PCR) assays and transcriptome analysis (RNA-seq) showed that the *PPR647* mutation significantly disrupted the expression of PEP-dependent plastid genes. In addition, RNA splicing and RNA editing of multiple chloroplast genes showed severe defects in *as-81647*. These results indicated that PPR647 is crucial for RNA editing, RNA splicing of chloroplast genes, and plays an essential role in chloroplast development.

## 1. Introduction

As the exclusive organelles for photosynthesis and energy transduction in plants, the chloroplast plays a vital role in plant growth and development. However, the process of chloroplast biogenesis and development is highly complex, and its molecular mechanisms have not been fully elucidated. Chloroplasts are semiautonomous organelles, which can encode 60–200 proteins, and their gene expression still undergoes regulation by the nuclear genome [1]. Nucleus-encoded polymerases (NEP) and plastid-encoded polymerases (PEPvc) are necessary enzymes responsible for plastid genes transcription and have significant impacts on chloroplast biogenesis [2,3,4]. According to the required transcriptase, chloroplast genes can be divided into three categories: Genes transcribed only dependent on PEP (mainly photosynthetic related genes, such as *psbA* and *psbD*); genes transcribed only dependent on NEP (mainly housekeeping genes such as *accD*, *rpoa,* and *rpob*); and genes transcribed by NEP and PEP (such as *atpE* and *16S rRNA* [2,3,4]). In addition, the expression of chloroplast genes is regulated by a series of post-transcriptional processes, including C-to-U RNA editing, intron splicing, and 5′- or 3′terminal maturation [5]. Mutants of those genes often resulted in leaf color variation [6,7,8].

Pentatricopeptide repeat (PPR) proteins are widely found in plants and play various functions in organellar metabolism. PPR proteins are characterized by tandem repeats of a highly degenerate 35 amino acid motif [9]. According to their tandem motifs, PPR proteins can be divided into two subfamilies: P-type and PLS-type proteins. Based on their different C termini, PLS-type PPR proteins can be further divided into E, E+, and DYW subgroups [10]. Plant PPR proteins are mainly located in chloroplasts or mitochondria [11,12,13]. Previous studies showed that most nucleus-encoded PPR proteins are involved in post-transcriptional gene regulation, such as RNA editing, RNA splicing, RNA stability, RNA translation, and RNA maturation [9].

Mutations in mitochondrial-localized PPR proteins are usually characterized by delayed plant growth, abnormal embryonic development, abnormal leaf shape, premature leaf senescence, and reduced seed yields [14,15,16,17,18,19]. For example, the PLS-PPR DEK36 in *Arabidopsis* and maize affected mitochondrial transcripts editing and seed development [15]. P-type PPR protein MTL1 affected the initiation of *NAD7* gene translation and delayed plant development [14]. Mutations of *ZmEMP18* and *OsPPR5* caused arrested embryo and early endosperm development by controlling the editing of mitochondrial *atp6*, *cox2*, and the cis-splicing of *nad4* intron 3, respectively [20]. Chloroplast-localized PPR proteins often affect chloroplasts development, resulting in plant leaf color variations. *ZmPPR4* is required for intron splicing of *rps12*, and the absence of *PPR4* can lead to an albino seedling-lethal phenotype [7]. *ZmPPR5* insertion mutant had viable embryos and was deficient in chloroplast ribosomes, eventually dying at the seedling stage [8]. Null alleles of PPR protein THA8 are seedling lethal in maize and embryo lethal in *Arabidopsis* [21]. *OSWLS4* is essential for chloroplast RNA group II intron splicing during early leaf development [22].

The reported chloroplast-localized PPR proteins in maize often affect the RNA editing efficiency or the intron splicing efficiency of some chloroplast genes, such as the splicing of *trnG* affected by PPR5 [6]; *rps12* requires PPR4 [8] and EMB2645 [23], and *ycf3* and *trnA* is affected by THA8 [21]. The editing of *atpA-1148* requires ZmPPR26 [24], and *rps8* is affected by ATP4 [25]. The maize genome encodes more than 600 PPR proteins. Most of their function remains unclear, and no chloroplast-localized PPR protein affecting these two functions at the same time has been reported in maize. To identify additional PPR proteins and elucidate their functions in organelles is vital for understanding plant growth and development. This study identified a novel PLS-type PPR protein, PPR467, which targeted the chloroplast. PPR467 is involved in RNA editing and splicing of plastid genes in developing leaves. Disruption of PPR467 function impaired chloroplast development and plastid gene expression and resulted in an albino-lethal phenotype. 

## 2. Results

### 2.1. Phenotypic Characterization of as-81647

**Figure 1:**
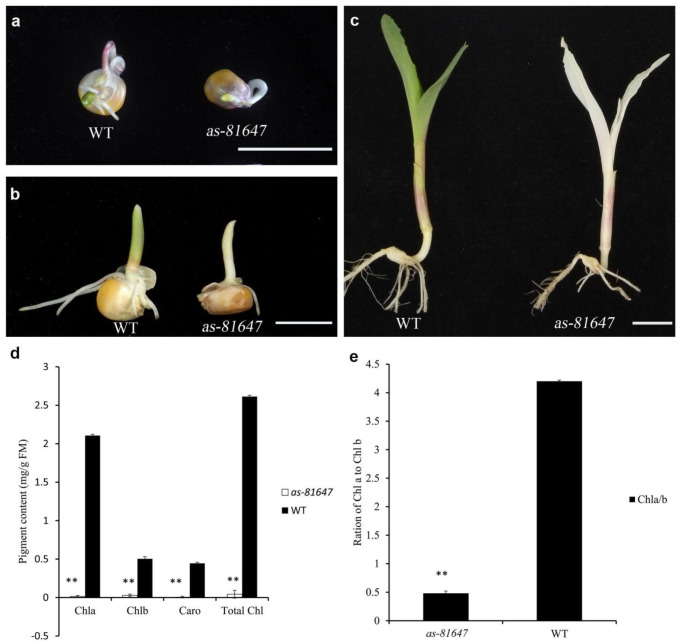
The phenotype of *as-81647*. (**a**–**c**) Phenotypes of *as-81647* after germination, at two days after planting (DAP) (**a**), 4 DAP (**b**), and 7 DAP (**c**). (**d**) Chl a, Chl b, Caro and total Chl content of WT and *as-81647*. (**e**) The ratio of Chl a to Chl b of WT and *as-81647*. Bar = 1 cm. ** *p* < 0.01 (Student’s *t*-test).

A lethal albino mutant was isolated from inbred line 81647 and designated *as-81647*. The *as-81647* mutant was albino from germination (Figure 1a–c) and only survived for about ten days. Consistent with the albino phenotype, the total chlorophyll content in *as-81647* was significantly lower than that in WT (Figure 1d). Detailed analysis revealed that the chlorophyll a (Chl a), chlorophyll b (Chl b), and carotenoid (Caro) contents of *as-81647* were only 5.8%, 1.64%, and 1.60% in WT plants. The ratio of Chl a to Chl b in *as-81647* was only 1/10 that in WT (Figure 1e). 

### 2.2. Abnormal Chloroplast Morphology in as-81647

**Figure 2:**
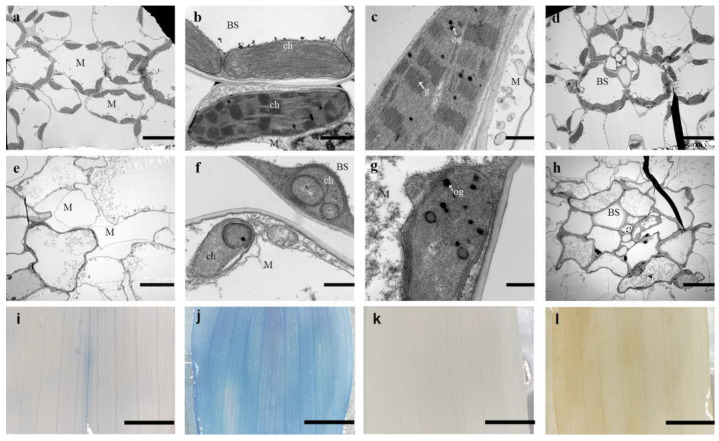
Electron microscopy observations of *as-81647.* (**a**,**e**) The number of chloroplasts per unit area in WT (**a**) and *as-81647* (**e**) plants. (**b**,**f**) Chloroplast structure in mesophyll cells and vascular bundle sheath cells in WT (**b**) and *as-81647* (**f**) plants. (**c**,**g**) Comparison of chloroplast structure between WT (**c**) and *as-81647* (**g**) plants. (**d**,**h**) Granum structure in WT (**d**) and *as-81647* (**h**) plants. *M* Mesophyll cell, *BS* Bundle sheath cells, *ch* chloroplasts, *g* granum thylakoid, *og* osmiophilic globule. Bar = 1 μm (**b**,**c**,**f**,**g**) and bar = 10 μm (**a**,**e**,**d**,**h**). (**i**,**j**) DAB staining of WT (**i**) and *as-81647* (**j**) leaves. (**k**,**l**) Trypan blue staining of WT and *as-81647* leaves. Bar = 1 cm.

We examined chloroplast structure from two-leaf stage seedlings of WT and *as-81647* by TEM (Figure 2a–h). Under normal conditions, chloroplasts in WT plants were crescent-shaped and contained well-developed thylakoid membranes consisting of stroma thylakoids and grana thylakoids (Figure 2a–d). In contrast, numerous vacuole cells without chloroplasts were found in *as-81647* (Figure 2e,h) and the remaining chloroplasts were small and severely deformed (Figure 2b,f). The granum lamellae were severely degraded with increased osmiophilic bodies and peroxides (Figure 2c,g).

### 2.3. Increased Reactive Oxygen Species (ROS) Levels in as-81647 

To explore the reason of cell death in *as-81647*, young leaves were stained with trypan blue and DAB (Figure 2i–l). Deep blue staining showed that the whole blade of *as-81647* was staining, while a few areas in WT (Figure 2j), indicating that a large number of cells died in the *as-81647* leaves. DAB staining showed a large amount of brown precipitate in *as-81647* (Figure 2k) but not in WT (Figure 2l), indicating excessive accumulation of H_2_O_2_ in *as-81647*. These results showed that the cells of *as-81647* leaves had died or were dying accompanied by the abnormal accumulation of ROS and that abnormal accumulation of ROS may be the direct cause of cell death in *as-81647*.

### 2.4. PLS-Type Pentatricopeptide Repeat (PPR) Protein PPR647, Is Responsible for the as-81647 Albino Phenotype

F_2_ populations derived from the crosses between B73 and *as-81647*/+ were used for genetic analysis and gene mapping. Among this monohybrid cross, all F_1_ plants had typical green leaves and approximately half of the F_1_ had phenotype-segregating ears. Moreover, the ratio of green to albino leaves in F_2_ offspring of the segregating ears was 3:1 (Table 1). This result suggested that *as-81647* is a recessive mutant.

**Figure 3:**
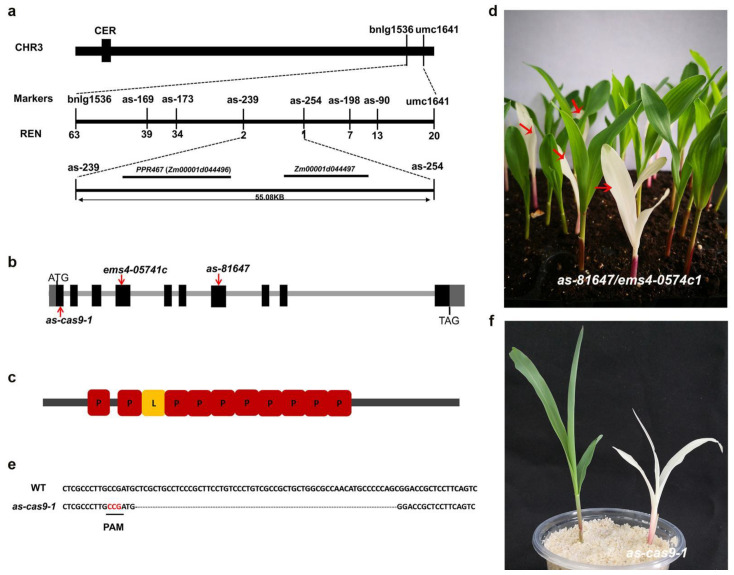
Map-based cloning of *as-81647*. (**a**) Gene mapping of *as-81647*. CER, Centromere. (**b**) The predicted motif structure of the PPR647 protein, P or P-L-S block motifs are depicted as boxes with letters as indicated; P, L designates PPR, PPR-L motifs, respectively. (**c**) Structure of PPR647 and the mutation sites within two alleles. Introns and exons were shown as solid lines and boxes, respectively. (**d**) Phenotypic analysis of the allelism test between +/*as-81647* and +/*ems4-05741c*. The red arrows indicate the mutant plants. (**e**) Sequence alignment of the edited sites of PPR647 in the first exon. PAM, adjacent protospacer motif. (**f**) The seedling phenotype of *as-cas9-1*.

An F_2_ mapping population from a cross between B73 and *as-81647*/+ was used to map the candidate gene responsible for the *as-81647* phenotype. By assaying 496 F_2_ albino mutants, we found bnlg1536 and umc1641 on the long arm of chromosome 3 were linked to *as-81647*, with genetic distances being 14.6 cM and 2.7 cM, respectively. Two extended populations with 840 and 1800 albino plants from different F_2_ populations were used for fine mapping, and the *as-81647* gene was ultimately narrowed down to a 55.08 kb region between markers *as-239* and *as-254* (Figure 3a). Two candidate genes (*Zm00001d044496* and *Zm00001d044497*) were identified in this region (Figure 3a). Sequencing analysis revealed four SNPs in these two adjacent genes. Further study found that the base substitution at +1034th base of *Zm00001d044496* resulted in an amino acid replacement (Asp to Lys). The base substitution at the second splice site of *Zm00001d044497* caused a Gly to Ser substitution.

To determine which gene was responsible for the *as-81647* phenotype, EMS mutant stock *ems4-05741c* of *Zm00001d044496* and *ems4-057444* of *Zm00001d044497* were obtained from the Maize EMS Stock Center (http://www.elabcaas.cn/memd; last accessed 8 September 2019). Phenotype analysis revealed that *ems4-05741c* exhibited albino leaves, and the offspring of (*as-81647*/+)/(*ems4-05741c*/+) showed a 3:1 ratio with green and albino leaves (Figure 3d), demonstrating that they were allelic. However, *ems4-057444* plants and the offspring of (*as-81647*/+)/(*ems4-057444*/+) all had green leaves. These results indicated that *ems4-05741c* could not complement the *as-81647* phenotype. *Zm00001d044496* is the target gene for *as-81647*. 

Another mutant allele of *Zm00001d044496* designated *as-cas9-1* was produced by CRISPR-Cas9. The gRNA spacer sequence in the first exon was selected as the target site for Cas9 cleavage (Figure 3e). *as-cas9-1* had a 58 bp deletion at the guide RNA (gRNA), created a premature stop codon in the mature transcript (Figure 3e). The phenotype of *as-cas9-1* homozygous produced similar albino seedling to *as-81647* (Figure 3f). These results further confirmed that *Zm00001d044496* is the target gene.

**Figure 4:**
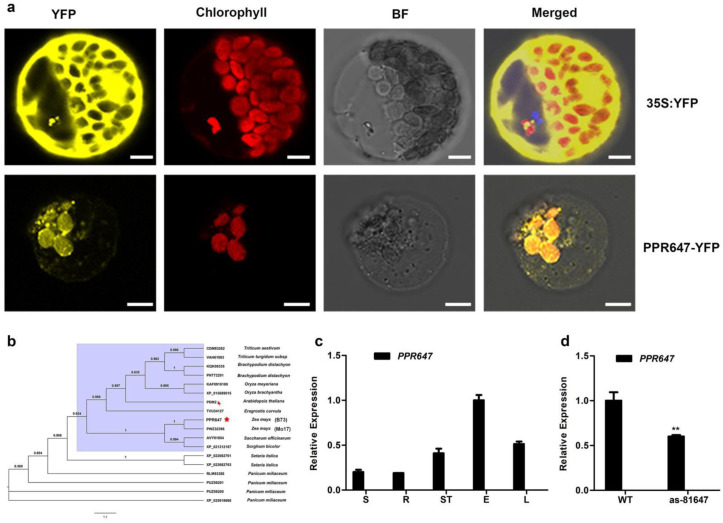
Gene expression pattern of PPR647. (**a**) Subcellular localization of PPR647 protein. bar = 5 μm. YFP, Yellow fluorescent protein, BF, Bright field. (**b**) Phylogenetic tree analysis of PPR647 protein. The red asterisk and arrows indicate PPR647 protein (in B73 background) and PDM2 protein, respectively. PWZ32396 is the PPR647 in Mo17 background. (**c**) The relative expression level of *PPR647* in various tissues was measured by qRT-PCR. RNA was isolated from the DAP20 seeds (S), roots (R), stem (ST), ears (E), seven-day leaves (L). (**d**) The relative expression level of *PPR467* in *as-81647* and WT at the three-leaf stage was measured by qRT-PCR. Values and bars represent the mean and standard obtained from three biological replicates, respectively. RNA level was normalized to that of the maize *ZmActin* gene (*Zm00001d010159*). Significant differences are indicated. ** *p* < 0.01 (Student’s *t*-test).

*Zm00001d044496* was predicted to encode a PLS-type PPR protein with 11 PPR motifs using Prosite, named PPR647 (Figure 3c). Among the 11 PPR motifs of PPR647, 10 are in tandem, and another one is interrupted by stretches of several amino acids. To examine the subcellular localization of PPR647, a vector containing 35S: PPR647-YFP was transiently transformed into maize protoplasts. As shown in Figure 4a, the YFP signal was co-localized with the chloroplast, indicating that PPR647 is a chloroplast-localized protein. Phylogenetic tree analysis showed that PPR647 was highly conserved in monocotyledons (Supplementary Figures S1 and S4b). The tree demonstrated that PPR647 is the orthologue of PDM2 of *Arabidopsis* [26]. Like *as-81647* albino seedlings, the seedlings of *pdm2* showed a pigment-defective phenotype which nicely supports the phylogenetic link [26]. To assess the role of *PPR647* in maize development, we examined its transcript expression pattern by real-time quantitative PCR (qRT-PCR) analysis, the result showed that *PPR647* was widely expressed in the root, stem, seed of DAP 20, ear, seven-day leaves (Figure 4c), consistent with the expression pattern shown in the publicly available Maize Gene Expression database (qTell) (https://qteller.maizegdb.org/; last accessed 6 October 2021) (Supplementary Figure S2). At the same time, we found that the expression of *PPR647* was significantly down-regulated in *as-81647* (Figure 4d).

### 2.5. PPR647 Mutation Affects Chloroplast-Associated Gene Expression during Leaves Development

**Figure 5:**
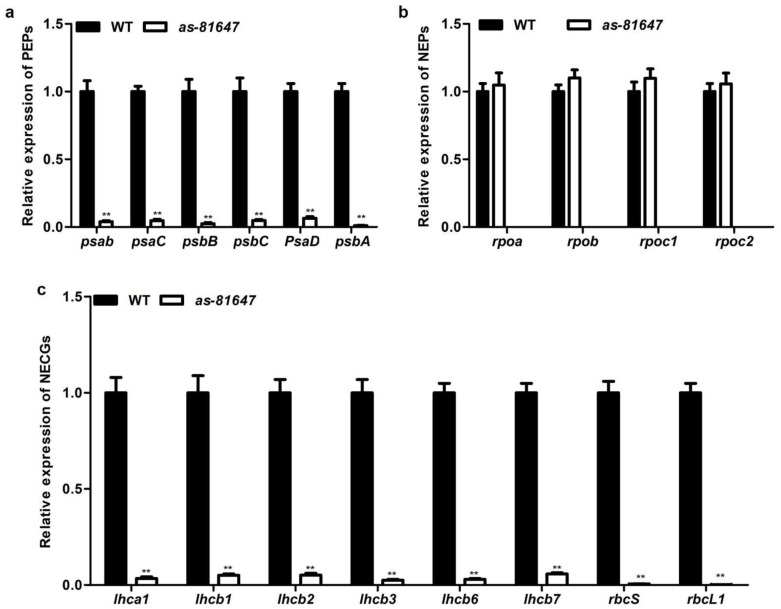
Relative expression levels of genes involved in chlorophyll biosynthesis and development. (**a**) qRT-PCR analysis of PEP-dependent photosynthesis genes (PEPs). (**b**) qRT-PCR analysis of NEP-dependent genes (NEPs). (**c**) RT-PCR analysis of photosynthesis-associated genes encoded by the nucleus (NECGs). Values and bars represent the mean and standard obtained from three biological replicates, respectively. RNA levels were normalized by the *ZmActin* gene (*Zm00001d010159*). ** *p* < 0.01 (Student’s *t*-test).

To explore the function of *PPR647*, qRT-PCR and RNA-seq analysis were conducted with the leaves at the two-leaf stage. Transcript levels of chloroplast-associated genes investigated by qRT-PCR results showed that the expression levels of all tested PEP-dependent photosynthesis genes (PEPs) were significantly downregulated (Figure 5a), while the expression of four tested NEP-dependent genes (NEPs) were slightly increased (Figure 5b). Expression levels of other photosynthesis-associated genes encoded by the nucleus were significantly reduced compared with WT (Figure 5c).

**Figure 6:**
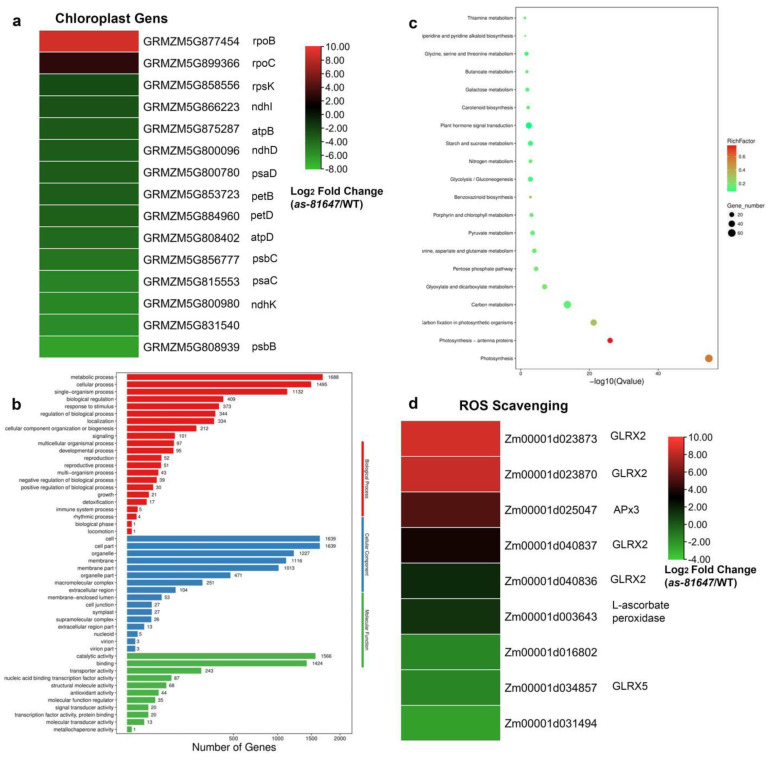
RNA-seq analysis of WT and *as-81647*. (**a**) Heat maps of differentially expressed chloroplast genes. (**b**) The most significantly related GO terms of the functional annotated DEGs, *p* < 0.05. biological process (Red box), cellular components (Blue box), and molecular functions (Green box). The x-axis represents gene number and the y-axis represents the GO terms name. (**c**) The top 20 enriched KEGG pathways. The x-axis represents the Q−value and the y-axis represents the pathway name. (**d**) Heat maps of differentially expressed genes involved in ROS scavenging.

From the RNA-seq analysis, 3473 DEGs were identified between WT and *as-81647* leaves, including 1766 up and 1671 down-regulated DEGs (Excel S1). Verification of the expression patterns of eleven DEGs via qRT-PCR revealed highly positive correlations between the RNA-seq data and qRT-PCR results (Table S2). According to the RNA-seq result, we found that most chloroplast genome genes were down-regulated, including PEP mediated genes and NEP, PEP co-mediated genes, while only NEP dependent genes were up-regulated (*rpob*, *rpoa*) (Figure 6c). Combining the results of RNA-seq and the qRT-PCR results led to the conclusion that the mutation of *PPR647* affected chloroplast-associated gene transcription, especially PEP mediated chloroplast genes.

Based on GO analysis, these DEGs were classified into different biological processes or molecular functions. In the cell components, DEGs were mainly concentrated in membrane parts, such as thylakoid, thylakoid membrane, photosynthetic membrane, chloroplast, chloroplast thylakoid, etc. In the biological process, DEGs were mainly enriched into photosynthesis, light reaction, generation of precursor metabolites and energy, and the oxidation-reduction process. At the molecular function level, the main functional items were oxidoreductase activity, tetrapyrrole binding, and chloroplast binding (Figure 6a). KEGG analysis showed that those DEGs were involved in 123 metabolic pathways, 28 of which were significantly enriched (corrected *p-value < 0.05*) (Figure 6b, Excel S2). Photosynthesis, carbon fixation, and carbon metabolism are the most significant enrichment pathways (Figure 6b). This result showed that PPR647 mainly affected the chloroplast development and photosynthesis of maize, consistent with the qRT-RCR result and albino phenotype.

Furthermore, expression levels of genes encoding ascorbate peroxidase and glutaredoxin (two major scavenger enzymes in the ROS degradation pathway) were mostly up-regulated (Figure 6d). This corresponds to the results of histochemical staining (Figure 2i–l). In *as-81647*, the increase of ROS production in cells activates the response of ROS scavenging genes. However, this response cannot balance the abnormal increase of ROS which eventually leads to cell death.

### 2.6. PPR647 Is Required for C-to-U RNA Editing of Multiple Chloroplast Transcripts

**Figure 7:**
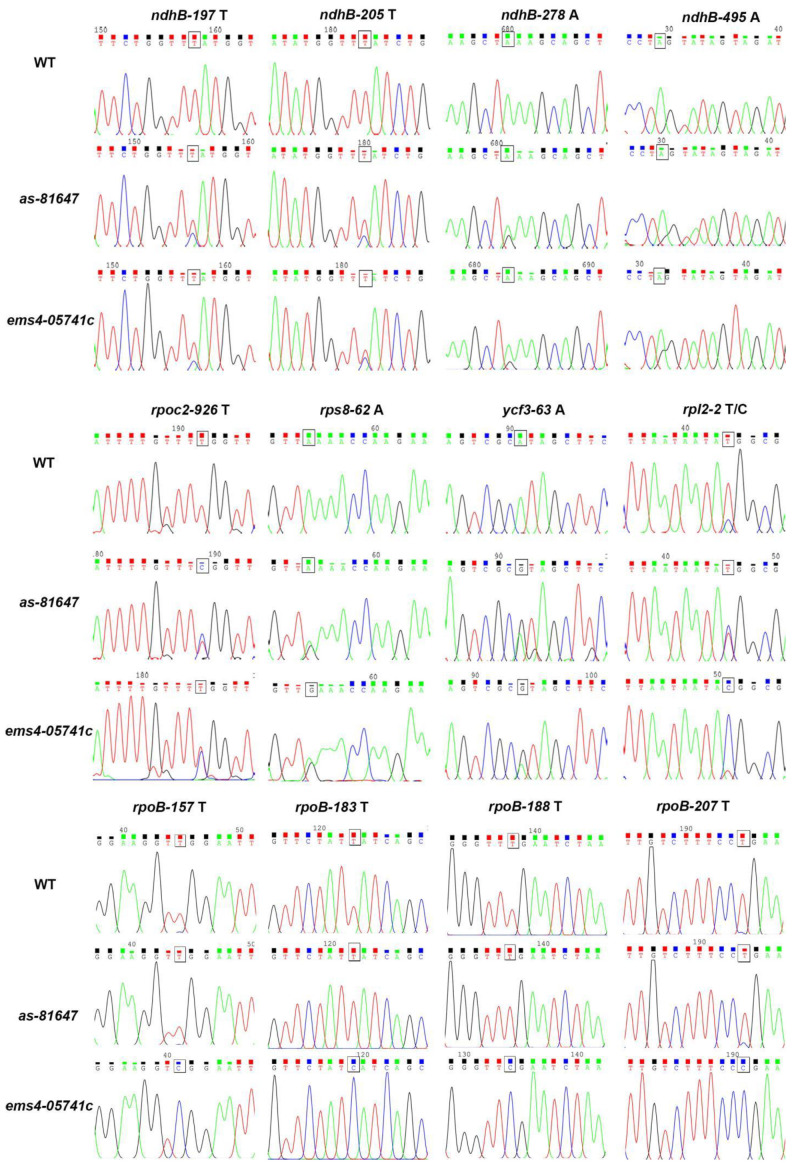
RNA editing analysis of chloroplast transcripts. The editing efficiency of multiple chloroplast transcripts was severely affected in *as-81647* and *ems4-05741c* plants. The box marks the editing site. T (A) stands for edited, C (G) stands for not edited, and T/C stands part edited in WT.

Accumulating evidence shows that PLS-PPR proteins are required for RNA editing [19,20,27]. To explore the function of PPR647, we investigated the editing efficiency of 27 identified RNA editing sites in maize chloroplast transcripts. The results showed that the editing efficiency of most sites was changed in *as-81647* and *ems4-05741c* (Figure 7, Supplementary Figure S3, Supplementary Table S2). Among them, the editing efficiency of *rpoc2-926*, *ycf3-63*, *rps8-62*, *ndhB-197*, *ndhB-205*, *ndhB-278*, *ndhB-495* sites, which were 100% edited in WT and significantly reduced in *as-81647* and *ems4-05741c* (Figure 7). In addition, all sites of *rpoB* were completely edited in WT and *as-81647* plants, but their editing efficiency was zero in *ems4-05741c* (Figure 7). For this phenomenon, we speculate that the different mutation sites of *as-81647* and *ems4-05741c* bring about inconsistent effects on *rpoB* editing. These data suggested that PPR647 is required for C-to-U RNA editing of multiple chloroplast transcripts.

### 2.7. PPR647 Affects the Splicing of rpl2 Transcripts in Chloroplasts

**Figure 8:**
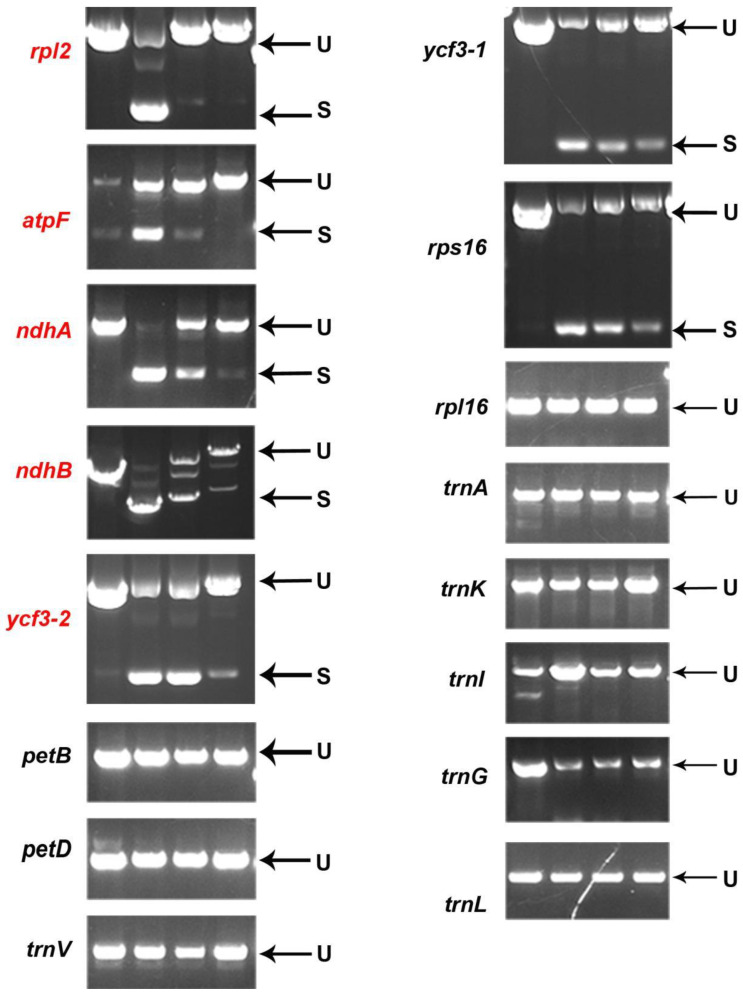
Splicing analysis of chloroplast transcripts in WT and mutants. From left to right, the template is DNA of WT, cDNA of WT, cDNA of *as-81647*, and cDNA *of ems4-05741c*. S, spliced; U, unspliced. The genes marked in red are genes affected.

To determine the function of PPR647 in RNA splicing of chloroplast genes, we performed RT-PCR analysis using primers located in exons flanking intron and then compared the lengths of the amplified products between WT and mutant plants. Nearly no mature *rpl2* transcripts were detected in *as-81647* and *ems4-05741c*, and compared with WT, the splicing efficiency of *atpF*, *ndhB*, *ndhA*, and *ycf3-2* significantly decreased in *as-81647* and *ems4-05741c* (Figure 8). This result indicated that PPR647 is essential for RNA splicing of chloroplast genes (especially the *rpl2* gene).

### 2.8. PPR647 Might Affect RNA Editing by Interacting with ZmMORF2

**Figure 9:**
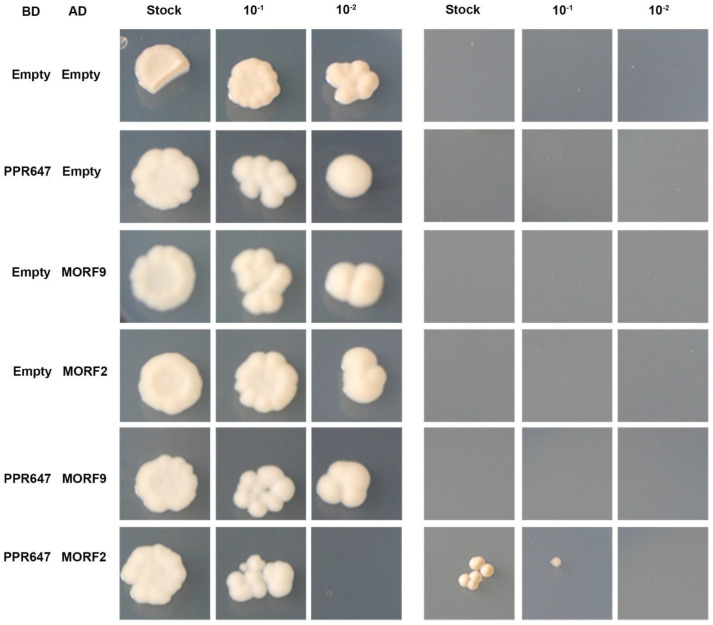
The interaction between PPR647 and MORF2 protein. Yeast two−hybrid assays indicated the interactions between PPR647 and MORF2. Proteins were obtained on the selective medium SD−Trp−Leu and SD−Trp−Leu−His−Ade. AD, activating domain; BD, binding domain.

Recent studies have shown that MORF2 and MORF9 are located in plastid and are required for chloroplast RNA editing [28]. To further investigate the role of PPR647 in RNA editing, we examined the physical interaction of PPR647 with ZmMORF2 (encoded by *Zm00001d026243*), ZmMORF9 (encoded by *Zm00001d024674*) using the yeast two-hybrid assay. From the result, we found PPR647 was determined to interact with ZmMORF2 but not ZmMORF9 (Figure 9), which suggested that PPR647 might affect RNA editing by interacting with MORF2. 

## 3. Discussion

There are hundreds of PPR proteins in maize, but only a few have been identified. Mutants of PPR proteins exhibit different phenotypes, such as leaf hypoplasia, growth restriction, photosynthetic pigment deficiency, and embryo or seed development defects [16,28,29,30,31]. In this study, a lethal albino seedling mutant (*as-81647*) was isolated and studied. In contrast to other reported albino mutants (*al1* and *al2*) [32,33], whose Chl deletion phenotype were transient, *as-81647* exhibits an albino-lethal phenotype throughout plant development. Corresponding to its phototype, we found that the chlorophyll content of *as-81647* is almost zero and most cells in *as-81647* had no chloroplast formation (Figure 2). Staining results showed that excessive reactive oxygen species accumulated in *as-81647* leaf and caused cell death (Figure 2i–l). Map-based cloning and transgenic analysis confirmed PPR647, a novel PLS-type PPR protein, is responsible for the albino phenotype of *as-81647*. Subcellular localization demonstrated it is function in chloroplasts (Figure 3). Studies have shown that the biogenesis of Chl is tightly with chloroplast development and its function [34]. So, those results indicated that *PPR647* may play an important role in maize chloroplast development.

PEP is a multisubunit polymerase that drives the transcription of photosynthetic genes in chloroplasts, such as *psbA*, *psbD*, *rbcL* [2]. NEP is a single subunit polymerase that responsible for the accurate transcription of plastid housekeeping genes, such as *rpoa*, *rpoB*, *rpoC1*, and *rpoC2* [2]. In our study, qRT-PCR and RNA-seq confirmed the transcription level of chloroplast gene mediated by PEP (PEPs) decreased significantly (Figure 5a) and genes mediated by NEP (NEPs) increased slightly (Figure 5b), suggested that *PPR647* is involved in regulating the gene expression of PEPs, and the reduced PEPs transcripts may be the reason of arrested chloroplast development in *as-81647*. Similar results have been established in previous studies, such as other PPR proteins AtACM1 [35], AtPDM3 [36] and OsWSL [37] in which the transcription levels of NEPs elevated and transcription levels of PEPs were decreased. In *acm1,* the accumulation of chloroplast rRNAs and ribosome subunit *RPS14* was disrupted, leading to the deletion of plastid ribosomes [35]. In *pdm3*, there is an increased steady-state level of *rpoB* transcripts but a reduced level of RpoB leading to a disrupted PEP complex [36]. In *wsl,* the low splicing efficiency of chloroplast transcript *rpl2*, leads to an aberrant transcript accumulation and the reduction of Rpl2 proteins, resulting in the lack of plastid ribosomes [37]. Chloroplast *rpl2* encodes the L2 subunit of 50S ribosomal protein, an essential component of the chloroplast ribosome, and the absence of this protein is a very sensitive marker for the absence of ribosomal function [38]. Splicing efficiency experiment showed that PPR647 dysfunction resulted in ineffective splicing of *rpl2* intron and reduced the splicing efficiency of *atpF*, *ndhA*, *ndhB*, and *ycf3.2* (Figure 8). RNA-seq data showed that the expression of these genes decreased in varying degrees, indicating that their transcription was affected (Supplementary Figure S4). Therefore, we speculate that the lack of plastid ribosomes is due to the ineffective splicing of *rpl2* and is one possible reason for the decrease of PEP-dependent transcript accumulation in *as-81647.* Multiple chloroplast genes (especially *rpl2*) require PPR647 to undergo splicing in maize.

The PEP complex consists of core subunits encoded by plastid gene (*rpoa*, *rpoB*, *rpoC1*, and *rpoC2)* and accessory protein encoded by nuclear gene (PAPs) [2]. Until now, many PAP mutants have been identified, such as *wls3*, *wlp2* in rice, *pap1*, *pap2*, *pap3*, *pap5*, *pap6*, and *pap10* in *Arabidopsis*, in which the transcription levels of NEP-dependent genes were also elevated and transcription levels of PEP-dependent genes were decreased [39,40,41,42,43]. At the same time, some proteins also caused this phenomenon due to their interaction with some proteins which directly interact with PEP complexes. For example, DG1 protein of *Arabidopsis* is involved in regulating PEPs transcription by interacting with SIG6 protein, which is necessary for PEP plastid gene transcription [44]. Therefore, another possibility is PPR647 may directly or indirectly bind to some components of PEP, that participate in the regulation of PEP transcription mechanism. Multiple chloroplast genes (especially *rpl2*) require PPR647 to undergo splicing in maize.

RNA editing, a post-transcriptional process, alters the RNA sequence by converting specific target cytidines in plastid transcripts to uridine. Our present study demonstrated that PPR647 affected the C to U editing efficiency of multiple plastid editing sites and interact with MORF2 (Figure 7, Supplementary Figure S3). Interestingly, we found that most of the affected sites were more influenced in *ems4-05741*, especially the *rpoB* (Figure 7, Supplementary Table S3). AS *ems4-05741* was a strong allele compared with *as-81647,* we speculate that the site affecting *rpoB* splicing in PPR647 may not be affected in *as-81647*. Sequence comparisons and phylogenetic analyses identified PPR647 as the orthologue of PDM2 from *Arabidopsi s* [26], which shows 46.3% sequence identity orthologue and 61.1% sequence similarity with PPR647. The *pdm2* mutant showed pigment-defective seedings, lower expression of all tested PEP-dependent transcripts, and higher expression of all NEP-dependent transcripts [26]. PDM2 is responsible for multiple RNA editing sites in plastid by interacting with MORF2 and MORF9. However, different from *as-81647*, embryogenesis was also affected in *pdm2*, and the splicing of plastid genes in PDM2 was not affected, indicated that there may be some functional differences between PDM2 and PPR647.

In summary, the maize chloroplast localized PPR protein PPR647 affected the transcription of PEP-dependent plastid genes, and simultaneously plays roles in both the RNA editing and intron splicing in plastid genes. Although the functions of many PPR proteins have been widely investigated, few of them simultaneously play roles in both RNA editing and intron splicing (except for rice WLS4 protein) [22]. Studies have shown that there is a feedback mechanism between intron splicing and RNA editing in chloroplast genome, altered editing is most likely an indirect effect of defective splicing of chloroplast introns in *as-81647*. At present, there is no PPR protein in maize that simultaneously affects the splicing and editing efficiency of chloroplast genes have been reported. As a PLS-family PPR protein, studies on PPR647 will help further explore the function of the PPR protein family and lay a foundation for revealing the relationship between RNA editing and splicing in plastid.

## 4. Materials and Methods

### 4.1. Plant Materials and Growth Conditions

The albino (*as-81647*) mutant was isolated from maize inbred line 81647. *ems4-05741c* and *ems4-057444* mutant lines were obtained from Maize EMS-induced Mutant Database (MEMD, http://www.elabcaas.cn/memd/; last accessed 8 September 2019) [33]. The preservation of *as-81647* depends on heterozygous plants (*as-81647/*+), and *as-81647*/+, used as the male parent, was crossed with B73 and YQ165 to generate F_1_ and F_2_. Albino plants in the F_2_ population were used for genetic mapping. All materials used for mapping were grown at the Experimental Station of Shandong Agricultural University in China. For other purposes, the plants were grown in growth chambers with a photoperiod comprising 16 h light at 28 °C (day), 8 h dark at 22 °C (night), and 70%~80% relative humidity. 

### 4.2. Photosynthetic Pigment Content Analysis

Approximately 100 mg fresh leaves of wild-type (WT) and *as-81647* plants were cut into pieces and placed into 95% ethanol for 48 h in darkness at 4 °C. After centrifugation, the supernatant was measured at 663 nm, 645 nm, and 470 nm with a UV-2450 instrument (Hitachi, Tokyo, Japan). Three biological repeats were measured for each sample. The pigment contents were calculated according to the following equations described by Arnon [45].

Chl a (mg/g) = [(12.7 × OD663 − 2.69 × OD645) × V]/(W × 1000)(1)

Chl b (mg/g) = [(22.9 × OD645 − 4.68 × OD663) × V]/(W × 1000)(2)

Caro (mg/g) = [OD470 × (V/W) − 3.27 × Chl a-104 × Chl b]/198(3)

### 4.3. Transmission Electron Microscopy (TEM)

Leaves from *as-81647* and WT plants were fixed with 2.5% glutaraldehyde in phosphate buffer (pH = 7.4) followed by osmium tetroxide and then dehydrated in an ethanol series before being infiltrated with Spurr’s resin. Polymerization was performed at 70 °C for 8 h. The specimens were sliced to yield ultrathin sections, stained with uranyl acetate and alkaline lead citrate before being examined with a JEM-1400Plus (JEOL, Tokyo, Japan) transmission electron microscope.

### 4.4. Histochemical Analysis

Trypan blue staining was used to detect cell death in leaves according to the methods of the previous study [46]. Fresh leaves were immersed in trypan blue solution (2.5 mg/mL trypan blue, 25% [*w*/*v*] lactic acid, 23% water-saturated phenol, 25% glycerol) at 70 °C for 10 min, then heated in boiling water for 2 min and incubated overnight at room temperature. Next, the sample was decolonized in a chloral hydrate solution (25 g in 10 mL of H_2_O) for three days. They were stored in 70% glycerol and analyzed with a stereomicroscope (Olympus szx12, Tokoyo, Japan). For DAB staining, leaf segments (approximately 5 cm in length) were immersed in DAB solution (1 mg/mL, pH = 3.8) for 8 h to react with the H_2_O_2_. They were placed in 75% ethanol, heated for 15 min and transferred to 10% glycerol for microscopic examination (Olympus szx12, Japan).

### 4.5. Map-Based Cloning and Allelism Test

Bulked segregant analysis (BSA) and simple sequence repeat (SSR) molecular markers were used for genetic mapping of the *as-81647* locus. For preliminary mapping, 308 publicly SSR markers distributed over the whole genome from MaizeGDB were used for polymorphism screening. For fine mapping, new markers designed by SSRHunter and Primer 6.0 were used. The PCR procedure was as follows: 95 °C for 5 min, followed by 35 cycles of 94 °C for 30 s, annealing for 30 s, 72 °C for 30 s, and a final elongation step at 72 °C for 10 min. The primer sequences are listed in Supplementary Table S1.

For the allelism test, *as-81647*/+ was crossed with *ems4-05741c*/+ and *ems4-057444*/+. Their hybrid progenies were planted, and their phenotypes were identified.

### 4.6. Generation of CRISPR-Cas9-Edited Mutant Alleles

The CRISPR-Cas9 vector of *Zm00001d004446* was constructed following a simplex editing strategy. The 20 bp target sequence (AAGCGGGAGGCAGCGAGCAT) for editing is located in the first exon of *Zm00001d004446*. Immature zygotic embryos (1.5–2.0 mm) of B104 were used for Agrobacterium-mediated maize transformation. T0 lines were hybridized to B104. For molecular identification of 15 F_1_ transgenic plants, a marker was designed to identify the editing effect of *Zm00001d004446*. The BAR gene was used to identify the stability of editing (the primers used are shown in Supplementary Table S1). The PCR products of the target were then cloned into a pMD18-T vector for sequencing analysis.

### 4.7. RNA Sequencing (RNA-seq) and Data Analysis

Total RNAs were extracted from the two-leaf stage leaves with three biological replicates (10 individuals per pool). RNA-seq libraries construction and sequencing were both performed on the Illumina HiSeq4000 platform at Novogene Bioinformatics Technology Co. Ltd. (Beijing, China). RNA-seq data were deposited in the National Center for Biotechnology Information (NCBI) Sequence Read Archive (SRA) under accession number SRP334160 (BioProject ID: PRJNA757925). After sequencing, low-quality reads were filtered to obtain clean reads, and these clean reads were then mapped to the reference genome (http://www.maizegdb.org/; last accessed 6 October 2021, Zea mays.AGPv4). Differential expression analysis of six samples was performed using the DESeq R package (version 4.1.1, U.S.A), and *p*-values were adjusted to control the false discovery rate. Unigenes with a revised *p (q) value < 0.05* identified by DESeq R were considered to be differentially expressed. GO annotation and GO enrichment analysis (corrected *p-value < 0.05*) of DEGs were performed to investigate their functions further. GO enrichment analysis of DEGs was conducted using GOseq R packages (version 2.1.2, USA) based on Wallenius non-central hypergeometric distribution [47]. GO terms with corrected *p (q) value < 0.05* were considered to be significantly enriched among the DEGs. Pathway-based analysis was conducted using KEGG [48].

### 4.8. RT-PCR and qRT- PCR Analysis

Total RNA was isolated from WT root, stem, leave, ear, developing seed at 20 DAP, and *as-81647* leave using an EasySpin plus Plant RNA Kit (Aidlab, Beijing, China). A HiFiScript gDNA Removal cDNA Synthesis Kit (CwBiotech, Beijing, China) was used to generate high-quality first-strand cDNA. RT-PCR was used to examine RNA splicing efficiency and RNA editing using specific primers as described previously. qRT-PCR was performed with cDNA dilutions to detect the expression of the target gene and verify the accuracy of sequencing using SYBR Premix ExTaqTM Kit (TaKaRa, Shiga, Japan) on an prism 7500 Real-Time PCR (ABI, Vernon, CA, USA) in a 20 µL reaction volume with 40 cycles. The primers used for RT-PCR and qRT-PCR are listed in Supplementary Table S1. The maize *ACTIN* (*Zm00001d010159*) gene was used as an internal control in the experiment.

### 4.9. Analysis of RNA Editing and Splicing of Chloroplast Genes

Specific primers were used to generate RT-PCR products covering each editing site, and the products were sequenced directly. RNA editing efficiency was estimated by the relative height of nucleotide peaks in the analyzed sequence. Specific primers used for chloroplast editing sites were quoted from the Hammani study [23]. RT-PCR was performed using particular primers situated in exons flanking the intron of each gene. Primers used for chloroplast editing and splicing are listed in Supplementary Table S1.

### 4.10. Phylogenetic Analysis

Homologous sequences were identified in NCBI (http://www. ncbi.nlm.nih.gov/; last accessed 6 October 2021) by performing a BLASTP search with PPR647 protein sequences. Amino acid sequences were aligned with MUSCLE in the MEGA7.0 software package using the default parameters. Evolutionary distances were calculated using Poisson correction analysis. The bootstrap method with 1000 replicates for phylogeny testing was used.

### 4.11. Y2H Assays

Full-length cDNA sequences of PPR647, ZmMORF2 (*Zm00001d026243*) and ZmMORF9 (*Zm00001d024674*) were cloned into the pGBKT7 and pGADT7 vectors (Promega, Madison, WI, USA). Constructs were subsequently co-transformed in pairs into yeast (strain AH109) following previously described methods [49].

### 4.12. Subcellular Localization of PPR647

The full-length CDS sequence of PPR647 without the stop codon was amplified by PCR from B73 and cloned into the transient expression vector pSAT6-EYFP-N1 to generate the fusion genes PPR647-YFP driven by the CaMv 35S promoter. The PPR647-YFP fusion product was introduced into Agrobacterium tumefaciens strain Gv3101 and introduced into maize protoplast as described. Fluorescence signals were detected using a Leica TCS SP5 II (Leica, Germany) laser scanning confocal microscope.

## 5. Conclusions

In this study, we identified a novel maize PLS-type PPR protein, PPR467, which is located within the chloroplast. Down-regulation of PPR467 impaired chloroplast development and PEP-dependent plastid genes expression and results in an albino-lethal phenotype. To summarize, PPR467 is functioning in chloroplast RNA editing, splicing, and is crucial for chloroplast development in maize.

## Figures and Tables

**Table 1:** Segregation of leaf color in the F_2_ population.

Number	Total	Normal Plants	Mutant Plants	Ratio of Segregation	χ^2^_0.05_ (1)
1	410	309	101	3/1	0.029
2	426	316	110	3/1	0.153
3	392	296	96	3/1	0.05
4	370	283	87	3/1	0.436
5	398	295	103	3/1	0.164

## Data Availability

The data presented in this study are available on request from the corresponding authors.

## Supplementary Materials

The following are available online at https://www.mdpi.com/article/10.3390/ijms222011162/s1. Figure S1: Alignment of amino acid sequences with the highest identity with the PPR647 protein. The *Arabidopsis* homolog is annotated as PDM2; Figure S2: The expression pattern of PPR647 from the publicly available Maize Gene Expression database (qTell). Different colors represent different processing conditions; Figure S3: RNA editing analysis of various target sites. The box is the editing site, T (A) stands for edited, C (G) stands for unedited, T/C stands part edited in WT; Figure S4: Relative expression of chloroplast genes which the intron splicing was affected in *as-81647* according to the RNA-seq dates; Table S1: Markers used in this research; Table S2: The expression value of 11 selected genes in RNA-seq and real-time PCR; Table S3: Editing efficiency of 27 sites in WT, *as-81647*, and *ems4-05741c*; Excel S1: The DEGs data of *as-81647* leaves vs WT leaves; Excel S2: KEGG enrichment of DEGs.
