# Supplementary material for: PPR647 Protein Is Required for Chloroplast RNA Editing, Splicing and Chloroplast Development in Maize
Source: Int J Mol Sci. 2021 Oct 16;22(20):11162. doi: 10.3390/ijms222011162 (PMC8537648; doi:10.3390/ijms222011162)
Supplement: Supplementary file 1 [file ijms-22-11162-s001.zip › Supplementary Figuer.pdf]

Article Title: PPR647 Protein Is Required for Chloroplast RNA Editing, Splicing and Chloroplast Development in Maize

Authors: Yan Zhao[1,2], Wei Xu[1], Yongzhong Zhang[1], Shilei Sun[1], Lijing Wang[1], Shiyi Zhong[1], Xiangyu Zhao[2], Baoshen Liu[1*]

Supplementary Figures

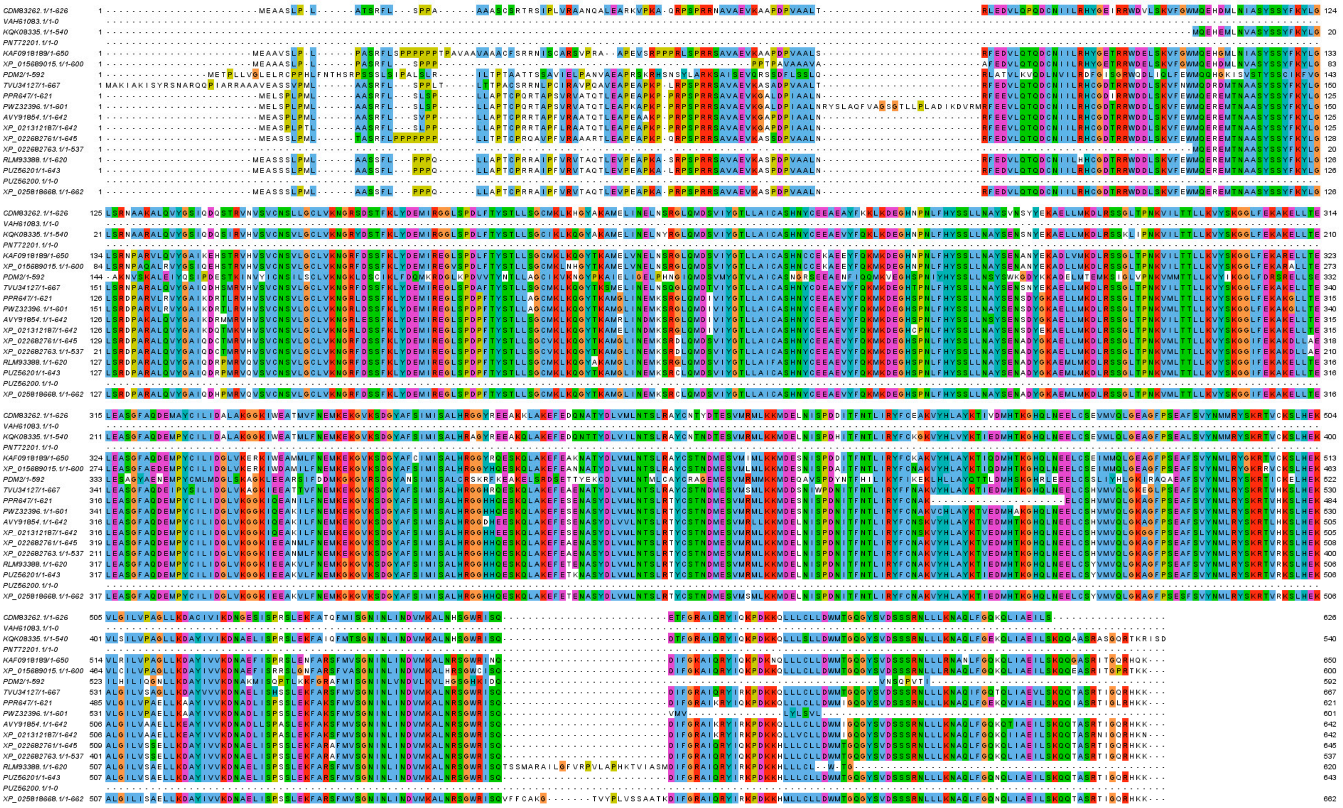

Supplementary Figure S1. Alignment of amino acid sequences with the highest identity with the PPR647 protein. The Arabidopsis homolog is annotated as PDM2.

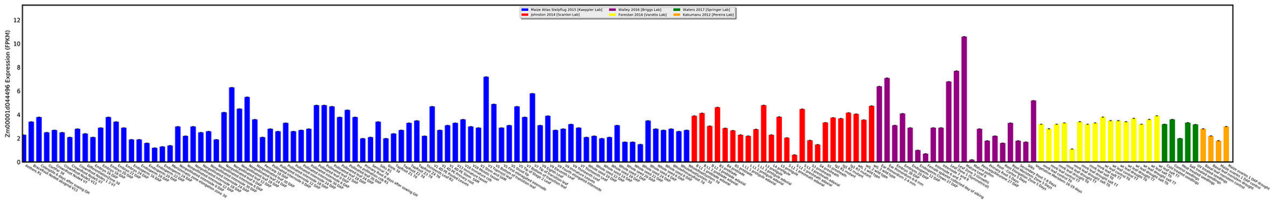

Supplementary Figure S2. The expression pattern of PPR647 from the publicly available Maize Gene Expression database(qTell). Different colors represent different processing conditions.

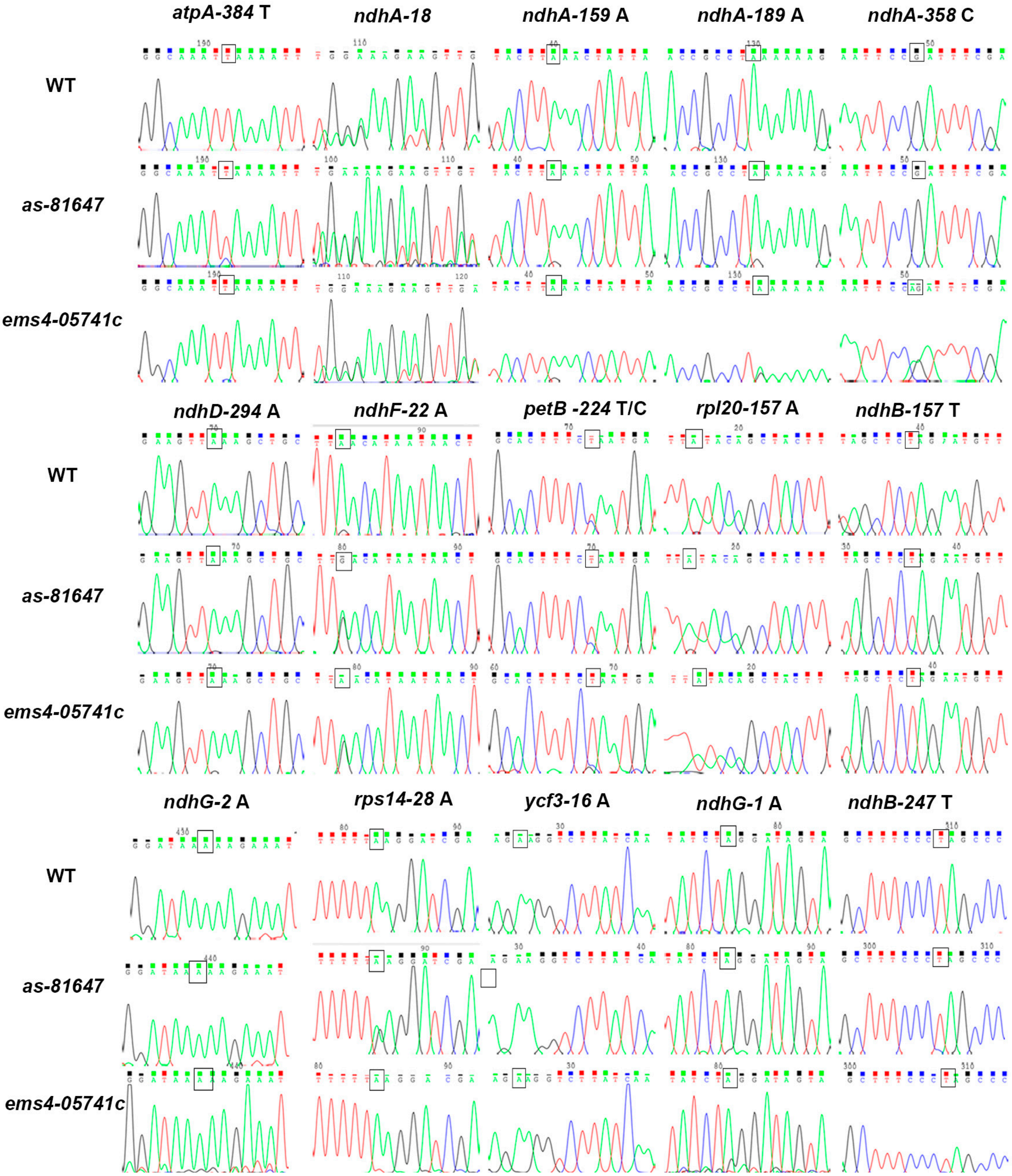

Supplementary Figure S3. RNA editing analysis of various target sites. The box marks the editing site. T (A) stands for edited, C (G) stands for unedited, T/C stands part edited in WT.

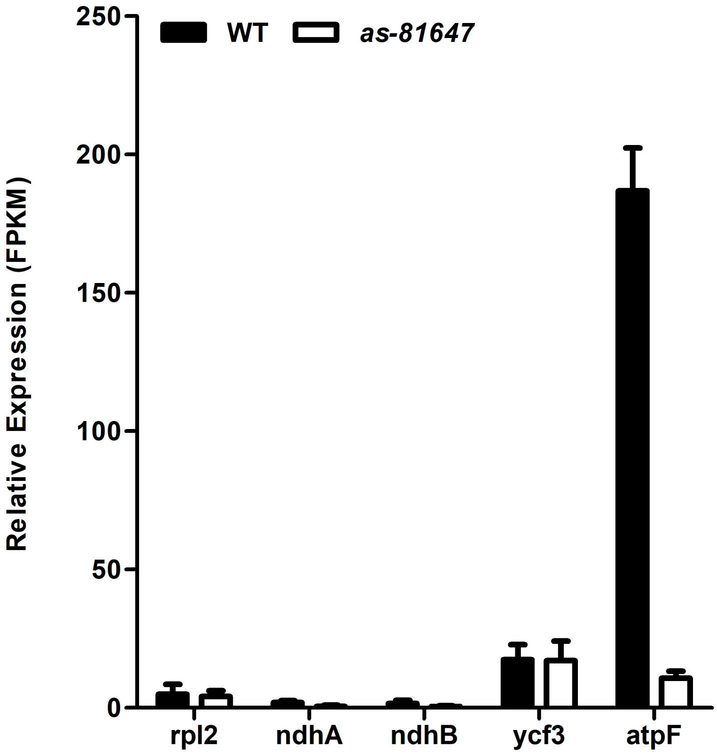

Supplementary Figure S4. Relative expression of chloroplast genes which the intron splicing was affected in *as-81647* according to the RNA-seq dates.
