# Supplementary material for: PPR647 Protein Is Required for Chloroplast RNA Editing, Splicing and Chloroplast Development in Maize
Source: Int J Mol Sci. 2021 Oct 16;22(20):11162. doi: 10.3390/ijms222011162 (PMC8537648; doi:10.3390/ijms222011162)
Supplement: Supplementary file 1 [file ijms-22-11162-s001.zip › Supplementary Table.pdf]

Supplementary Table S1–S3

Article title: **PPR647 Protein Is Required for Chloroplast RNA Editing, Splicing and Chloroplast Development in Maize**

Authors: Yan Zhao[1,2], Wei Xu[1], Yongzhong Zhang[1], Shilei Sun[1], Lijing Wang[1], Shiyi Zhong[1], Xiangyu Zhao[2], Baoshen Liu[1*]

Table S1. Markers used in this research.

Marker	Forward primer (5'–3')	Reverse primer (5'–3')	Experiment
umc1536	TGGTTCTTGGACCGTACGTAAATC	AAAAACGAAATTTGTTAGGCCAGG	
umc1641	CTCCCTTCGTCTCCCGACTC	CAGATCGGCTCAGCCACAAC	
as-30	ATATGGGCTCGGGAAGCT	AGTGACGATGGATGTGAACG	
as-90	CTCTTGGCGTGCTTCTTCCTG	GCCGACATGCTGGCGTTC	
as-160	AAGCCAAGCGATCCACAAG	TCGGAAGAGCACGCAAGAA	For gene mapping
as-173	TTTTTTTGTTGCCCACCCA	TGCCCCGGCCTAACTATCT	
as-198	AGAAGGCAGGAAGAGGGGG	TTTGGAGAATGGCGGGTG	
as-239	AATTAGATGTAGCAGGGCCAAG	ACCATTCGTATCATTCAGGGG	
as-254	TTCTAAAAATTTCTGGCTCCG	CTTATATCAATATGGTTTCCCTCG	
PP-1	AGTCGTGAACGCAAACAGAT	TTCACAAGAGAATGCAAAGGT	
PP-2	GCGTTGGGATGACCTCTCTAAG	CAAAAATCATGCCCTCCTGG	
PP-3	TGAAGAATGGGAGGTTGGAC	ATTATGGCTGTAGCTAGATGGG	
PP-4	AGAGTTTGATTTTTTTTCCTTC	CCTTTTTCTTTCATTTCGTT	
PP-5	TTTTCTGAGGTCCTTATCTTATTT	TATCTTCAACTGTTTTGTATGCTAG	For *Zm00001d044496* sequencing
PP-6	AGTATAGCTTGAGCTTGTCGAAT	CAATTTGATGTGCTTAGTGTGG	
PP-7	ATACTAACCTATTCTTCCTTCCAACA	ATCTTGTCTCTAACTTACCCCTCA	
PP-8	AGACACAAAAAATGATAAAGGAAAGAT	CCTTCAGACAACACAATCTAACACAC	
PP-9	ATCCTTTTGTTTTGCAGCCC	AATCATAGAGTCCCCACCGTTC	
PP-10	TCAGATTATGGTATGCGGTAT	CTTCAAGCCTTTCAATAGAGAT	
PC-1	ACCATCTTTTGTTCACGCCTTT	TTGCCTTCCTTCTCTCATTTTTT	
PC-2	ATGTGCTCAAAAGTCAAGCTAAAAG	CAAAACCACTGTCTCAGAAAATCAA	
PC-3	TATCCCTTTGTAAACCCCCAG	GTTAAATCAACCCGCATCATT	
PC-4	TTTTTTCAACTCCCAGCCTCTT	TTGCTCTTCATACCTTTCTCCCT	
PC-5	ACAAGCAGCACGAAGCAATAAG	GGAACAAGCCCGTCAAGAAT	For *Zm00001d044497* sequencing
PC-6	GAAGGACAAGGAAAATAAAAGC	TTGAAGTATAGCAACGTGATGAA	
PC-7	AAAGACTAAAATAAAGGAGAAAGAAG	CAGTATACAGTACAATAGGCGGTG	
PC-8	GCCTTCGTTTTGGAGATTTTAGA	TGGATGATTGCACTTTGGTTTT	
PC-9	TTTTTCAACTCCCAGCCTCTT	TTTGGCAACCCCATTTTTCC	

PC-10	ATCGTCTTGTCGGTTTTCGGA	TGATTGCACTTTGGTTTTGGC	
rpoB	ACCGCTCGGAATTAGACCATAAGGG	CCAGGTCCCCACCTACACAAGC	For chloroplast editing analysis
rpoC2	TCGACTAGGTGGATTGGTCCGAGT	ATCCTGCGGGAAAAGTGTTGCT	
atpA	AGGCGAAGGGAGTATGACCGCT	CAGAGCGGAGGCGAATTGTGC	
rps14	CGTCGATGAAGGCGTGTAGGTGC	CAGAGGGAGAAGAAGCGGCAGA	
ycf3-2	GGGGTTTCGTTCTAGCGCCCG	GGATGTTGGCTCAATCCGAAGGAA	
ycf3-1	CCCTTTTTTCCCCGGAGGTTGTCG	ATGCCTAGATCCCGTATAAATGGAAAT	
rpl20	ATTGATGATCCTTATTTTATTGGAAATCGTG	CGGATAAACGCAGCAACACGGG	
petB	GGAAGTGCTAGTGTGGGCCAATCC	CTCTATGCTATGCCTTCCCTATAACGGACC	
rps8	AGCCAGGTCGGCTGATACGCT	ATGGGCAAGGACACTATTGCTGATTT	
rpl2	TTCCCTTGCGTGTGCTCGGG	CGTATGATCATTACCCTTCAACCGGG	
ndhF	GACAGGTGAAGTGAGAATACCATAGC	TGGAACATACATATCAATATGCCTGGG	
ndhD	CCAATGTTGGTTATGGAACCAATTCC	GGAATTGTTACCGCATGCTCATT	
ndhG	GATAAAAATCGGTTGCTAAATGAATCCC	GTCAGTTCATGAAAAATTTTATACTAGAAATTTC	
ndhA-1	CTAATGAACCCAGTTAGCATAGGGAAC	GGACTTTACCCAGGATGAGAATGGATCAG	
ndhA-2	CCTATAGGCTGACGCCAAAGATTCCATCC	CAAGTATTGCTCCTATTGGTCTTCTCATGG	
ndhA-3	CGAGTACTTCTATTGTGATTCCCAGTAGG	AGATATCATTATGGGGGAAGTTGATCG	
ndhB-1	GTATTAACAGCTACTCTAGGGGGAATG	TCCTTCGTAGACGTCAGGAGTCC	
ndhB-2	CCCCCACTCCAGTCGTTGCTT	CCTAGCAGCTAAAAGAGGGTATCCTG	
425	GAGTCGTGAACGCAAACAGATAG	GTCACGGCTCAGTCCAAGGT	For identifying the editing effects of *Zm00001d004446*
Zm00001d019605	GCCTACGCCCCATAGAAACT	TGCCATACTCCCAGGAAAAG	For qRT-PCR
Zm00001d053015	CGGAGCAAGTAGCCGAGTA	ATGGCGTTCAGGTTCTGG	
Zm00001d025103	ATCACCGGCTTGGACATAAG	TCCATCTGGAACTCCCAATC	
Zm00001d011847	CCTGGAGAGGAGGAAGAAGG	AGGCGGAGGAGTAGGTTGAT	
Zm00001d018159	GCATCTGGCTCTGTTCCTC	CGTTCTGCTTCCACGACTC	
Zm00001d025059	GTGCGTGTGAATGGGTTTC	CGACCTTGTTGTTGGTGTCC	
Zm00001d004380	GTGACCGAGTTTTCCTGTGG	GGAGTTCAGAGAGGGTGTCG	
Zm00001d011833	CTCAAACTTCCTGTGTGACTTG	GAGCATGATGACTGTTGCATTG	
Zm00001d034543	ATTGTAATTTGGCTGCTGGTAC	GTCGATAGGCGTTATGTTATGC	

Zm00001d026599	CGAGAACTTCGCTAACTTCAC	AATCGATAACTGACTCGGTCG
Zm00001d019147	ACTGCCACGTTGTATATAAGCC	CATAGCTGGGTGAGTGAAGG

Table S2. The expression value of 11 selected genes in RNA-seq and qRT-PCR.

Genes	RNA-seq		qRT-PCR		Gene Function
	WT	*as-81647*	WT	*as-81647*	
Zm00001d019605	1	0.007	1	0.078	NADH-plastoquinone oxidoreductase
Zm00001d053015	1	0.006	1	0.003	uncharacterized protein
Zm00001d025103	1	0.043	1	0.067	primary amine oxidase-like
Zm00001d011847	1	0.000	1	0.002	putative HLH DNA-binding domain superfamily protein
Zm00001d018159	1	5.497	1	3.068	alpha-amylase precursor
Zm00001d025059	1	285.754	1	3.523	Germin-like protein subfamily 1 member 8
Zm00001d004380	1	0.024	1	0.283	10-deacetylbaccatin III 10-O-acetyltransferase
Zm00001d011833	1	0.004	1	0.005	ferredoxin--NADP reductase
Zm00001d034543	1	0.138	1	0.019	photosystem I reaction center subunit II
Zm00001d026599	1	0.243	1	0.019	light harvesting chlorophyll a/b binding protein 6
Zm00001d019147	1	2.795	1	1.838	40S ribosomal protein S2-like

Table S3. Editing efficiency of 27 sites in WT, *as-81647* and *ems4-05741c.*

Editing sites	WT (%)	*as-81647* (%)	*ems4-05741c* (%)
Rpoc2-926-T	100	41	55.3
Rps14-28-T	100	53.6	100
AtpA-384-T	100	75.9	97.8
Rpob-157-T	100	94.4	0
Rpob-183-T	100	100	0
Rpob-188-T	100	100	0
Rpob-207-T	100	86.5	0
PetB-224-T/C	60	74.2	90.9
Rps8-62-T	100	56.7	55.5
Rpl2-2-T/C	78.9	43.9	10.6
Ycf3-16-T	100	100	100
Ycf3-63-T	100	39	32.5
Rpl20-104-T	100	100	100
NdhD-294-T	100	97.6	95
NdhF-22-T/C	94.2	54.3	54.7
ndhG-1-T	100	93.4	82.9
ndhG-2-C	0	0	12.8
ndhA-18-T/C	57.1	65.8	68
ndhA-159-T	100	90	80
ndhA-189-T	100	91.5	65
ndhA-358-C	0	0	51.4
ndhB-157-T	100	100	100
ndhB-197-T	100	66.7	77.5

ndhB-205-T	100	60	62.1
ndhB-247-T	100	92.1	92.3
ndhB-278-T	100	63.6	63.9
ndhB-495-T	100	80	67.9

T stands for edited, C stands for not edited, T/C stands part edited in WT.
