# Supplementary figures and images for: PPR647 Protein Is Required for Chloroplast RNA Editing, Splicing and Chloroplast Development in Maize
Source: Int J Mol Sci. 2021 Oct 16;22(20):11162. doi: 10.3390/ijms222011162 (PMC8537648; doi:10.3390/ijms222011162)

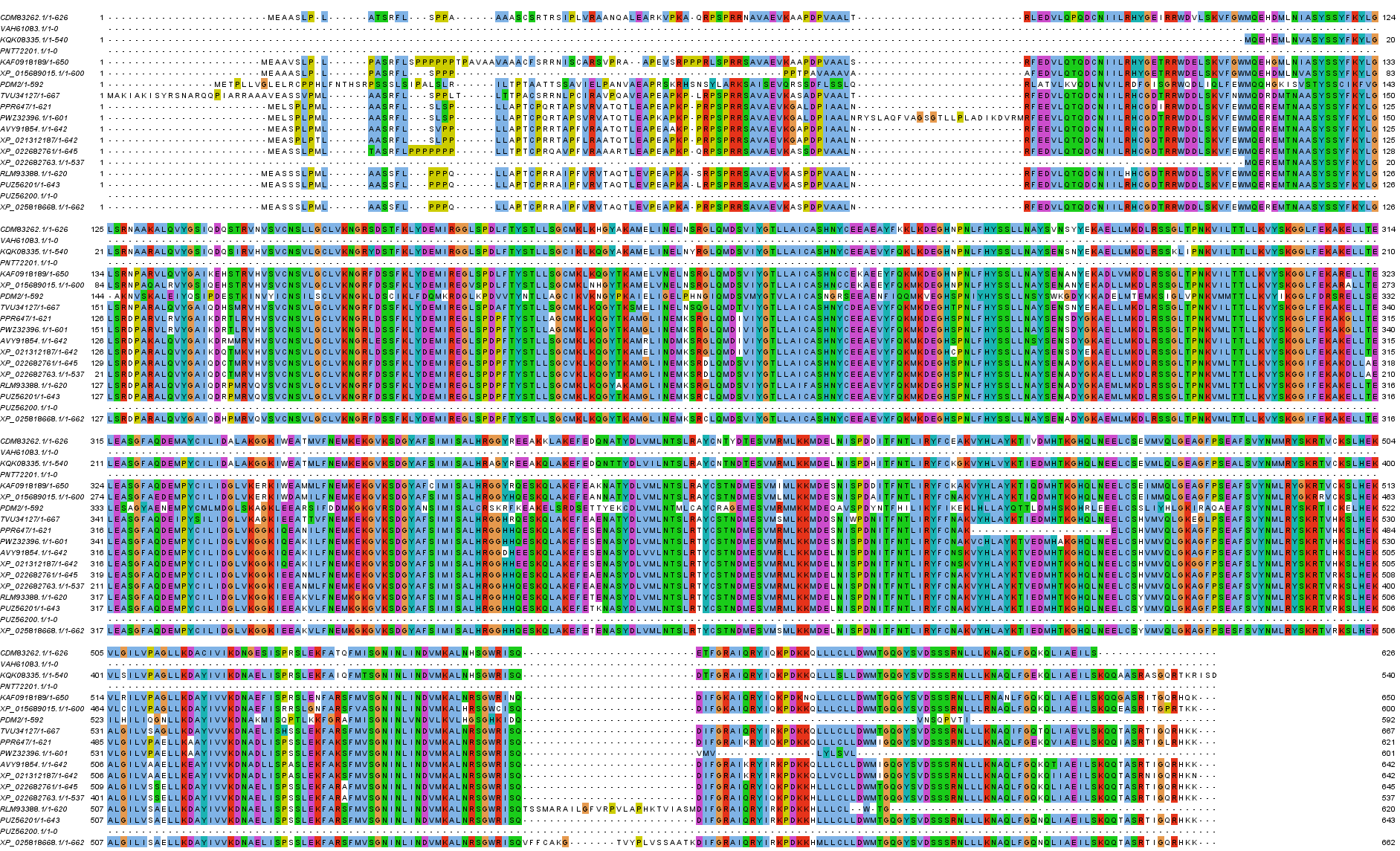

Supplement: Supplementary file 1 [file ijms-22-11162-s001.zip › Fig.S1.JPG]

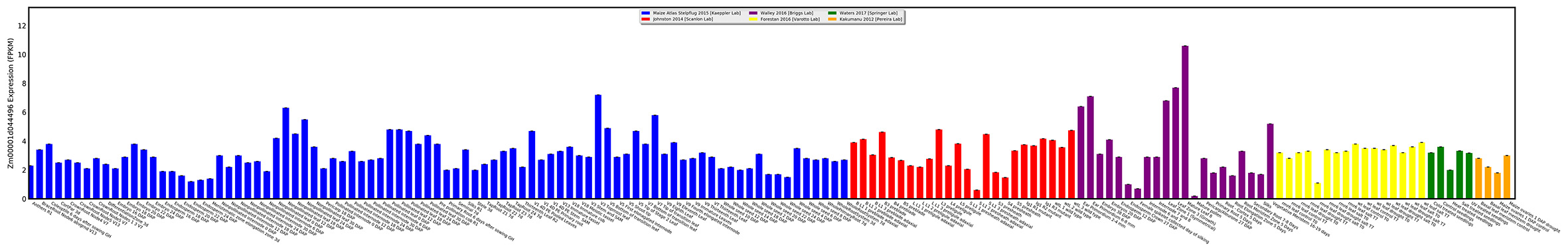

Supplement: Supplementary file 1 [file ijms-22-11162-s001.zip › Fig.S2.jpg]

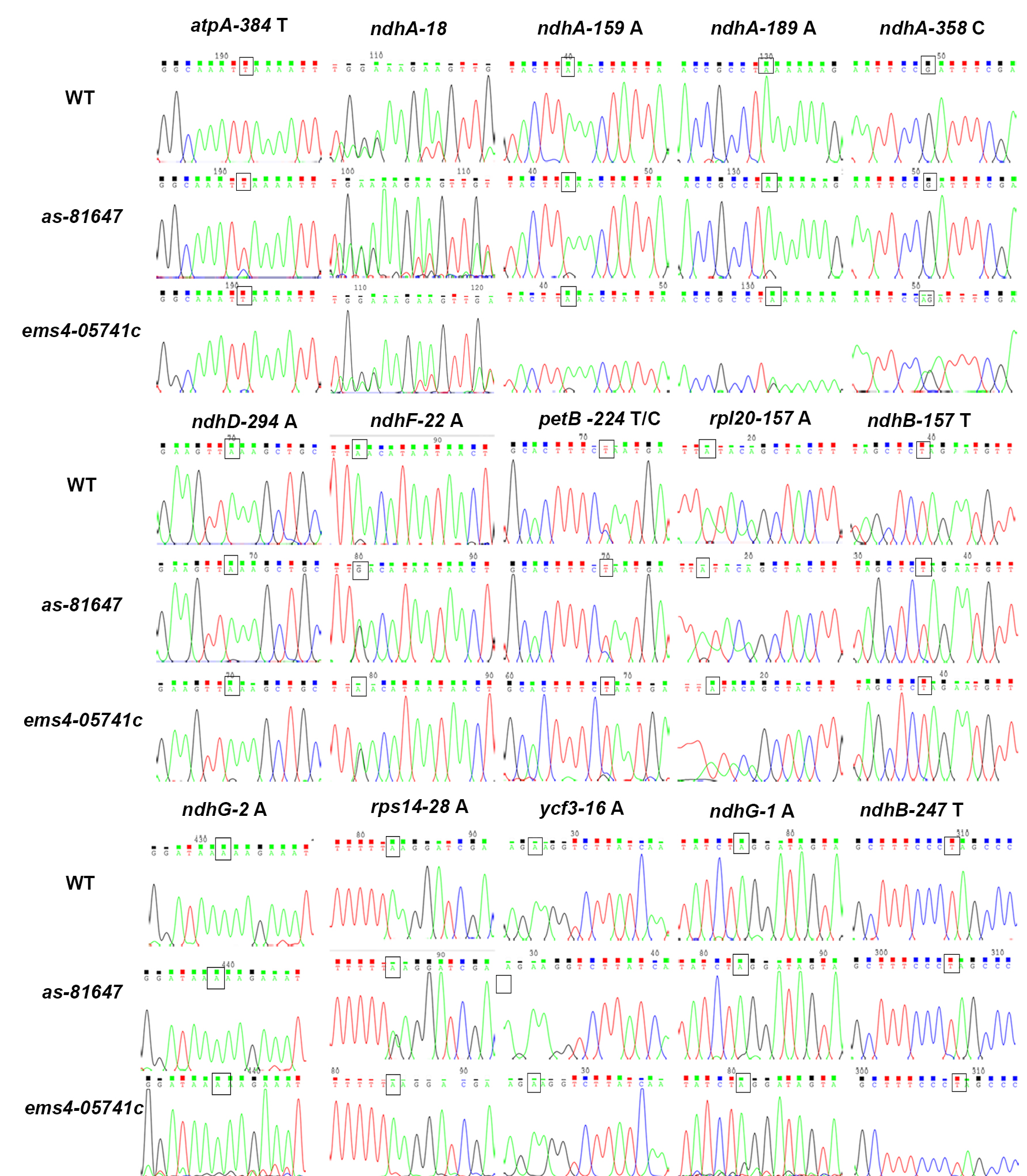

Supplement: Supplementary file 1 [file ijms-22-11162-s001.zip › Fig.S3.jpg]

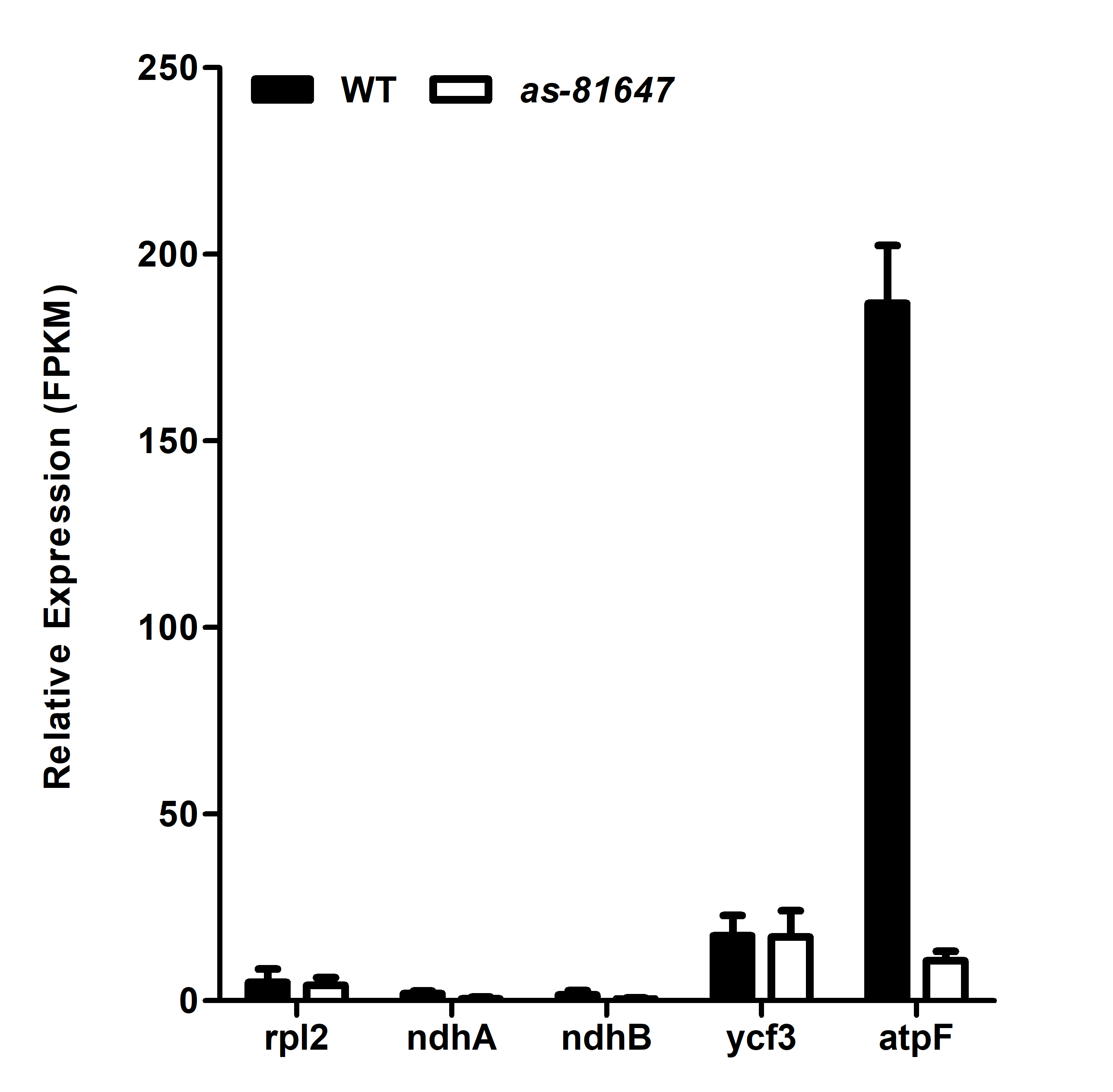

Supplement: Supplementary file 1 [file ijms-22-11162-s001.zip › Fig.S4.jpg]
